# Possible involvement of Toll-like receptor 8-positive monocytes/macrophages in the pathogenesis of Sjögren’s disease

**DOI:** 10.3389/fimmu.2024.1480675

**Published:** 2024-10-31

**Authors:** Lijing Yan, Yuka Miyahara, Mizuki Sakamoto, Naoki Kaneko, Hu Chen, Junsei Sameshima, Hajime Kido, Shiho Yokomizo, Tomoki Sueyoshi, Haruki Nagano, Yukiko Ohyama, Seiji Nakamura, Shintaro Kawano, Masafumi Moriyama

**Affiliations:** ^1^ Section of Oral and Maxillofacial Oncology, Division of Maxillofacial Diagnostic and Surgical Sciences, Faculty of Dental Science, Kyushu University, Fukuoka, Japan; ^2^ Oral Health, Brain Health, Total Health (OBT) Research Center, Faculty of Dental Science, Kyushu University, Fukuoka, Japan; ^3^ Section of Oral and Maxillofacial Surgery, Division of Maxillofacial Diagnostic and Surgical Sciences, Faculty of Dental Science, Kyushu University, Fukuoka, Japan; ^4^ Faculty of Dental Science, Kyushu University, Fukuoka, Japan

**Keywords:** Sjögren’s disease, Toll-like receptor 8, monocyte, macrophage, CD86

## Abstract

**Background:**

Sjögren’s disease (SjD) is an autoimmune disease marked by lymphocytic infiltration of salivary and lacrimal glands, leading to glandular dysfunction, where CD4-positive helper T (Th) cells and their cytokines are crucial in the pathogenesis. Recent studies have demonstrated that Toll-like receptors (TLRs), particularly those recognizing immune complexes containing DNA and RNA, contribute to Th cell activation in various autoimmune diseases. This study explores the expression and function of these TLRs in SjD.

**Methods:**

DNA microarray analysis of salivary gland tissue from six SjD patients and real-time PCR (n = 32) was used to identify overexpressed TLRs. Single-cell RNA sequencing (scRNA-seq) was performed using tissue lesions and integrated with published scRNA-seq data from tissues and peripheral blood mononuclear cells to examine gene expression in macrophages and monocytes. Finally, multi-color immunofluorescence staining was conducted to confirm TLR8 expression and function in SjD lesions (n = 19).

**Results:**

DNA microarray analysis revealed the up-regulation of *TLR8*, along with other TLRs and innate immune response genes in SjD. Real-time PCR showed significant up-regulation of *TLR7* and *TLR8*. *TLR8* up-regulated in both analyses. In scRNA-seq analysis, the *TLR8*-expressing cluster comprised macrophages and monocytes, which also produced T cell activation genes like *CD86*. TLR8-positive macrophages infiltrated inflammatory sites and frequently expressed CD86 in quantitative imaging approaches.

**Conclusions:**

These results suggest that infiltrating monocytes and macrophages may produce cytokines and chemokines through TLR8 stimulation, potentially enhancing B7 molecule expression, promoting the adaptive immune response, and contributing to SjD pathogenesis.

## 1 Introduction

Sjögren’s disease (SjD) is a systemic autoimmune disease characterized by the lymphocytic infiltration of affected glands, concomitant destruction of glandular tissue, and autoantibody production (1). The mechanisms involved in the pathogenesis of SjD are unclear; thus, the use of systemic immunosuppression has inconsistently shown improvement in sicca symptoms, and no definitive treatment has been established (2). As with other autoimmune diseases, studies on the pathogenesis of SjD focused mainly on aspects of adaptive immunity. In our previous study, we showed that striking infiltration of cluster of differentiation (CD)8^+^ cytotoxic T lymphocytes and a prominent proportion of apoptotic cells in primary SjD could be accounted for by epithelial cells, including ductal and acinar cells, by a quantitative approach (3). In comparison, the relevance of innate immunity, which induces an immune response in advance of adaptive immunity, to the pathogenesis of SjD is not well studied.

The relevance of Toll-like receptors (TLRs), which are pattern recognition receptors enrolled for pathogen recognition and a pivotal factor in innate immune responses, in the pathogenesis of systemic autoimmune diseases is a subject of intense research and is being studied by many rheumatologists and researchers (4, 5). In diseases such as systemic lupus erythematosus, immunoglobulin (Ig) G4-related disease and SjD, where there is an abundance of DNA and RNA-containing immune complexes, TLRs targeting these complexes including TLR7, TLR8, and TLR9 underscore their critical contribution to disease pathogenesis (4, 6). We previously reported that M2 macrophages promote fibrosis via up-regulated TLR7 signaling in IgG4-related disease, which shares commonalities with SjD, such as glandular enlargement, sicca symptoms, and circulating autoantibodies (6, 7). In SjD model mice, TLR7 signaling drives the development of SjD via interferon (IFN)-α and tumor necrosis factor (TNF) expression (8, 9). Considering the existence of TLRs such as TLR8, which are thought to function differently in humans and mice, and their eventual clinical application, human subjects should also be investigated. The issue of whether these TLRs for detecting DNA and RNA-containing immune complexes are directly related to the pathogenesis of SjD, specifically the initiation or progression process, remains unclear. If these TLRs does contribute to the pathogenesis, the question arises: Which of the above TLRs is a causative trigger?

A better understanding of the pathogenesis of any disease related to the immune response with tissue involvement, including SjD, is likely best obtained by a detailed and quantitative investigation of disease lesions. We have previously used a quantitative imaging approach to elucidate immune responses in a variety of diseases, including SjD, IgG4-related disease, tumors, and coronavirus disease 2019 (3, 10–12). In this study, we found striking expansion of TLR8-expressing monocytes/macrophages, which produce T cell activation chemokines/cytokines in SjD, using DNA microarray analysis, single-cell RNA-sequencing (scRNA-seq) analysis, and quantitative analysis of multi-color immunofluorescence (IF) staining. Our results provide a comprehensive landscape of the dynamics of the initiation and progression mechanisms in SjD and indicate that TLR8 might be pivotal to the pathogenesis of SjD and a potential future therapeutic target.

## 2 Method

### 2.1 Study participants

Labial salivary gland (LSG) biopsy tissues from six patients with SjD and three patients with jaw deformity, as well as three unaffected submandibular gland (SMG) tissues obtained through neck dissection in patients with oral squamous cell carcinoma were used in the DNA microarray analysis. Tissues from 32 SjD patients and 18 healthy donors were obtained for real-time PCR testing. LSG tissue from a patient with SjD was used for scRNA-seq analysis after homogenization. LSGs from 19 patients with SjD and SMGs from 6 patients with chronic sialadenitis (CS) were stained using multi-color IF.

All SjD patients presented with various degrees of symptoms of salivary gland involvement, lymphoid infiltration in biopsy tissues, and positive results for anti-SSA/Ro or anti-SSB/La antibodies, in accordance with the 2016 American College of Rheumatology/European League Against Rheumatism classification criteria (13). Only untreated primary SjD patients were enrolled in this study, and none of the patients present with a medical history of lymphoma or cryoglobulinemia. All patients received no concomitant therapies at the time of history taking, clinical examinations and pathological tissue biopsy. A summary of patient information is presented in **Supplementary Table 1**.

This study was approved by the Ethics Committee of Kyushu University (IRB number: 29-639), and all tissues were obtained from the Department of Oral and Maxillofacial Surgery, Kyushu University Hospital. The patients or their immediate family members provided fully informed written consent before being enrolled in the study. The methods were performed in accordance with the approved guidelines.

### 2.2 Sample preparation for DNA microarray analysis

Samples were classified by disease condition into an SjD group, which comprised LSGs from six SjD patients, and a healthy control (HC) group, which comprised LSGs from three patients with jaw deformity and uninvolved SMGs from three oral squamous cell carcinoma patients. Using protocols recommended by Invitrogen (San Diego, CA, USA), total RNA was extracted using TRIzol^®^ Reagent (Invitrogen) and purified using the SV Total RNA Isolation System (Promega, Madison, WI, USA). Total RNA was quality checked using the Agilent 2200 TapeStation System (Agilent Technologies, Santa Clara, CA, USA). Labeling reactions were performed with a Low Input Quick Amp Labeling Kit, one-color (Agilent Technologies) using 50 ng of total RNA for each labeling reaction. In accordance with Agilent’s recommended protocols, hybridization was performed using the SurePrint G3 Human Gene Expression v2 8 × 60 K Microarray Kit (Agilent Technologies) (DNA chip including 60,000 probes), followed by washing and scanning using a SureScan Microarray Scanner (Agilent Technologies). The output from the scanner was then converted to numerical values considered as background using Feature Extraction Software ver. 9.5.1.1 (Agilent Technologies).

### 2.3 Data pre-processing and analysis

For the digital outcomes of the DNA microarray analysis, the quantile normalization of the expression was performed using the limma v3.54.2 package in R (www.r-project.org) (14). Principal component analysis was performed for linear dimensionality reduction. To address the batch effects, harmony v0.1.1 in R (15) was used to eliminate the implications then uniform manifold approximation and projection was used for data visualization (16). Permutational multivariate analysis of variance analysis was also performed to compare the significant differences between SjD and HC groups using non-parametric tests, which was done through the adonis function of the vegan package v2.6-4 in R (17). The normalized expression data without batch effect correction was used to calculate differentially expressed genes (DEGs) via the edge package v3.40.2 in R (18), with the criteria of log 2-fold change > 1.5 and adjusted *P*-value < 0.05.

### 2.4 Gene enrichment analysis

DEGs were used for the gene enrichment analysis, including gene ontology (GO) analysis (19) and Kyoto Encyclopedia of Genes and Genomes pathway analysis (20), to determine whether any of the genes attributed to the pre-defined sets for the GO terms and Kyoto Encyclopedia of Genes and Genomes pathway were present in DEGs. Additionally, gene set enrichment analysis was performed to sort the list of all detected genes by rank score, which was defined as −log10 (*P*-value) * log 2-fold change. The data were analyzed using the clusterProfiler v4.7.1.002 package in R (21, 22).

### 2.5 Real-time PCR

Data for 32 SjD patients and 18 HCs were included in the real-time PCR analysis, which was performed using the Brilliant III Ultra-Fast SYBR^®^ Green qPCR Master Mix (Agilent Technologies), as follows: 10 µl of Master Mix, 10 ng of template DNA, and 0.5 µl of 20 pM sense and antisense primers were added to sterile water for a total reaction volume of 20 µl. The reaction conditions were as follows: the first cycle of thermal denaturation was performed at 95°C for 5 minutes; second and subsequent cycles for 10–20 seconds; and the extension reaction at 72°C for 10– 30 seconds. Primers for *TLR7*, *TLR8*, and *TLR9* are listed in **Supplementary Table 2**. To quantify the messenger (m)RNA expression levels between cases, the expression levels of each mRNA were corrected using the mRNA expression level of the housekeeping gene, *β-actin*, to calculate the relative expression levels. Statistical analysis was performed using the Mann–Whitney U test, with *P* < 0.05 considered statistically significant.

### 2.6 Tissue homogenization and library construction for scRNA-seq

Tissue homogenization and library construction were performed in accordance with a previously described method (23).

### 2.7 Quality control and scRNA-seq analysis

Several published datasets (24, 25) were integrated with our dataset, including the tissues of 11 SjD patients and 5 with HC, as well as the peripheral blood mononuclear cells (PBMCs) of 5 SjD patients and 5 HCs. The analysis tools were centrally based on the Seurat package v5.1.0 (26) in the R v4.2.2 environment and scvi-tools (27) in the Python (www.python.org) environment. A variational autoencoder-based deep learning model scVI (28) was used to remove known batch effects while clustering the cells by the top 4000 highly variable genes and to compute the coordinates for visualization. For immune cell phenotypes, assigning was performed by the marker genes, as follows: *CD79A*, *CD27*, and *XBP1* for antibody-secreting cells; *CD19*, *MS4A1*, and *CD79A* for B cells; *CD3E*, *CD4*, and *IL7R* for CD4^+^ T cells; *CD3E*, *CD8A* for CD8^+^ T cells; *ITGAX*, *FCER1A*, and *CST3* for dendritic cells (DCs); *CD14*, *CD68*, and *AIF1* for macrophages/monocytes (Mφ/mono); *NCAM1*, *FCGR3A*, *GNLY*, and *NKG7* for natural killer cells/natural killer T cells; *FCER1A* for mast cells; and *CLEC4C* and *IL3RA* for plasmacytoid DCs. Cells labeled with Mφ/mono were processed for gene expression analysis. Specifically, these cells were then divided into two groups, *TLR8* ^+^ or *TLR8* ^−^, on the basis of *TLR8* expression, for the analysis.

### 2.8 Multi-color IF staining and cell quantification

The staining and quantification steps were described previously (23). The following primary antibodies were used: anti-CD68 (1:400, #76437, Cell Signaling Technology, Danvers, MA, USA) for macrophages; anti-TLR8 (1:100, ab228962, Abcam, Cambridge, UK); anti-CD80 (1:200, ab134120, Abcam); anti-CD86 (1:100, #91882, Cell Signaling Technology); anti-CD8 (1:200, ab85792, Abcam, Cambridge, UK); and anti-CD4 (1:500, ab133616, Abcam, Cambridge, UK). The TLR8^+/−^ macrophages in the SjD and CS tissues, as well as CD86/CD80^+^ TLR8^+/−^ macrophages in SjD tissues were counted to calculate absolute numbers and proportions. Statistical analyses were performed using GraphPad Prism 9 (GraphPad Software, Inc., San Diego, CA, USA). Comparisons between groups were performed using the Mann–Whitney test and Wilcoxon matched-pairs signed rank test, considering *P* < 0.05 as statistically significant.

## 3 Results

### 3.1 Investigation of TLRs and expressing cells via microarray, scRNA-seq, and IF staining

**Figure 1:**
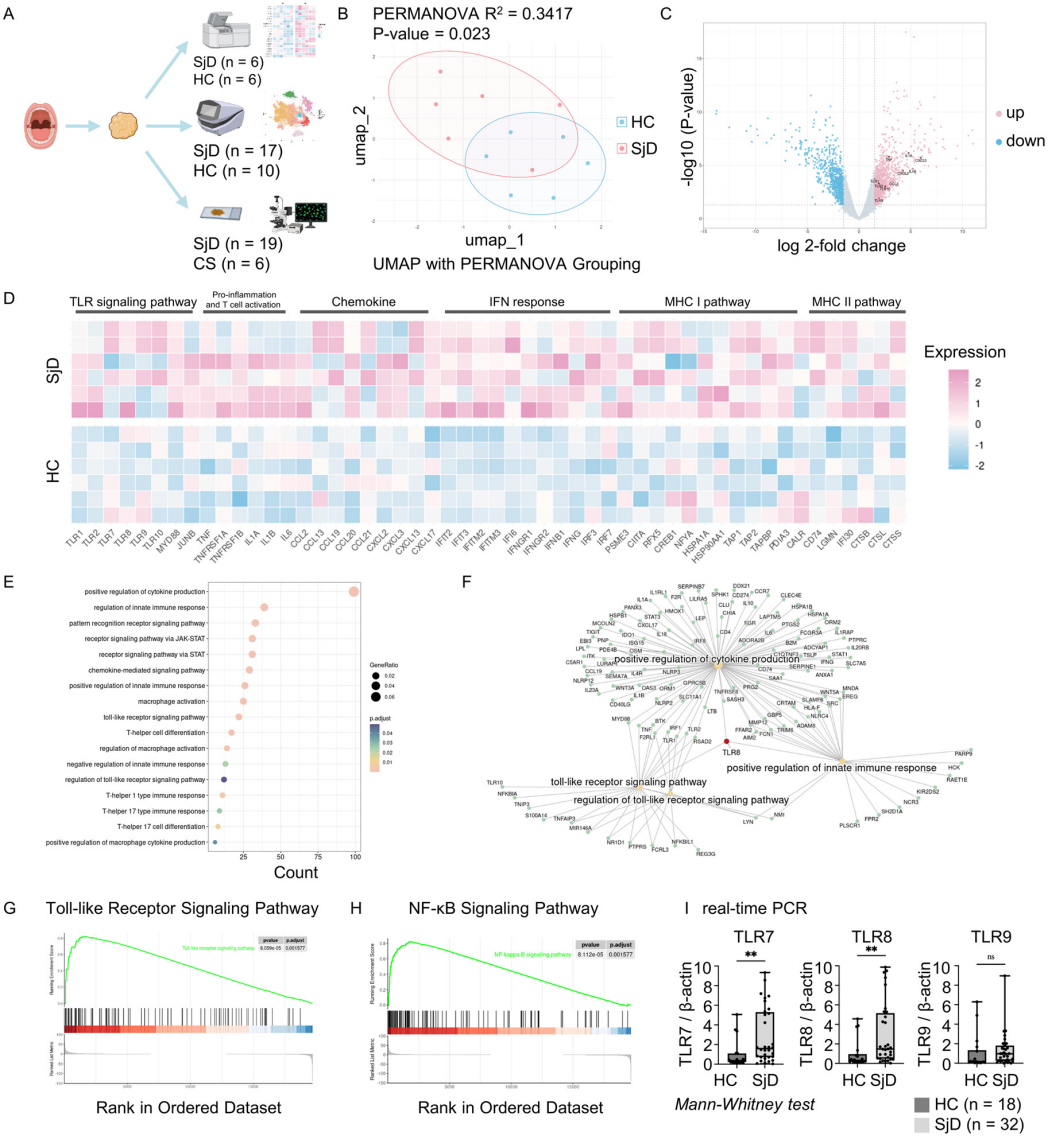
Workflow and microarray analysis. **(A)** Summary and schema of the workflow. Salivary gland tissue and PBMCs were used for the DNA microarray and scRNA-seq analyses, and multi-color immunofluorescence staining. Figure created with BioRender.com. **(B)** UMAP visualization and PERMANOVA analysis. PERMANOVA was computed based on coordinates derived from the UMAP after removing batch effects. R^2^ indicated 34.17% variation between the SjD and HC groups, with a *P*-value of < 0.05 indicating a significant difference. **(C)** DEGs of SjD compared with HCs. Genes were computed using the edge package in R for the microarray data. Cutoff values were set at log 2-fold change > 1.5 with an adjusted *P*-value of < 0.05. The DEGs counts were 996 up-regulated and 743 down-regulated among a total of 22,173 genes. **(D)** Heatmap for DEGs between the SjD and HC groups. The selected genes for presentation revealed a different gene expression pattern between SjD and HC regarding the TLR signaling pathway, pro-inflammation, chemokines, IFN response, and MHC I/II pathways. The order of the samples in each group was sorted in ascending order of a monocyte marker CD14 expression, from top to bottom. **(E)** GO enrichment analysis based on DEGs. Enriched gene ontology terms for DEGs in the SjD group compared with the HC groups are presented as a dot plot, in which the dot size represents the gene ratio and color represents the *P*-values (all, < 0.05). **(F)** Linkages of genes and GO categories visualized via a Cnetplot. *TLR8* plays a central role in the connecting TLR signaling pathway, positive regulation of cytokine production, and positive regulation of the innate immune response. **(G, H)** KEGG pathway gene set enrichment analysis. TLR and NF-κB signaling pathways were significantly up-regulated in the SjD group, with adjusted *P*-values of 0.0016 for both. **(I)** Real-time PCR for specific TLRs. *TLR7* and *TLR8* were significantly up-regulated in the SjD group (n = 32) versus the HC group (n = 18), determined by Mann–Whitney *U* test (**P < 0.01; ns, not significant). PBMC, peripheral blood mononuclear cell; scRNA, single-cell RNA; UMAP, uniform manifold approximation and projection; PERMANOVA, permutational multivariate analysis of variance; SjD, Sjögren’s disease; HC, healthy control; DEGs, differentially expressed genes; TLR, Toll-like receptor; IFN, interferon; MHC I/II, major histocompatibility complex I/II; GO, gene ontology; KEGG, Kyoto Encyclopedia of Genes and Genomes; NF-κB, nuclear factor-κB; PCR, polymerase chain reaction.

The roles of TLRs in the pathogenesis of autoimmune diseases have been widely studied (4). To understand the relevance between these TLRs and SjD, as well as identify which types of TLRs drive autoimmune responses in SjD patients, we processed LSG biopsy samples from six SjD patients, three non-SjD patients, and SMG biopsy samples from three patients with oral squamous cell carcinoma, in the microarray analysis. We also homogenized an LSG biopsy sample from an SjD patient and performed scRNA-seq on this sample. After quality control and integration with other published scRNA-seq analysis data, we constructed a transcriptional dataset with 84,017 immune cells from 17 SjD patients and 10 healthy donors with both PBMCs and tissue samples. Multi-color IF staining and quantification of the staining images were also performed on 20 LSG tissue samples from patients with SjD and seven SMG tissue samples from CS patients (**Figure 1A**).

### 3.2 Microarray analysis revealed the activation of innate immune responses in SjD

After removing the potential batch effect using the harmony algorithm, we computed UMAP for each sample and performed permutational multivariate analysis of variance analysis based on these coordinates (**Figure 1B**). The results revealed a statistically significant difference between the SjD and HC groups (*P* = 0.023; R^2^ = 0.3417). We then analyzed the DEGs between the two groups (**Figure 1C**). *TLR8* and other TLR family genes, such as *TLR1*, *TLR2* and *TLR10*, as well as pro-inflammatory cytokines and chemokines, such as *IL1A*, *IL1B*, *TNF*, *CCL2*, *CXCL2*, and *CXCL3* were up-regulated in the SjD group. The heat map also demonstrated the different expression patterns of genes related to pro-inflammation and activation of innate immune responses between the SjD and HC groups (**Figure 1D**). To achieve an overview of the features in the SjD group, we performed GO enrichment analysis on these DEGs (**Figure 1E**). The GO enrichment analysis in biological processes shows the enriched terms, such as positive regulation of cytokine production, macrophage activation, T cell responses, and regulation of innate immune responses. In this study, the GO enrichment analysis revealed the potential immune response processes in SjD tissues. Intriguingly, *TLR8* was pivotal in the positive regulation of cytokine production, the TLR signaling pathway, and positive regulation of innate immune responses (**Figure 1F**), emphasizing the necessity of further investigation of *TLR8* and its expressing immune cells.

### 3.3 Up-regulation of TLR and the nuclear factor-κB (NF-κB) signaling pathways in SjD

Innate immune responses are always accompanied by the activation of certain signaling pathways. To more accurately study the signaling pathways up-regulated in SjD, we performed gene set enrichment analysis on the microarray data (**Figures 1G, H**, **Supplementary Figures 1A, B**). The TLR signaling pathway and NF-κB signaling pathway showed statistically significant up-regulation in the SjD group compared with the HC group (adjusted *P*-value of 0.0016 for both), indicating the potential activation of an innate immune response and the pivotal role of TLRs in this response. We also used real-time PCR to determine the expression of mRNA for TLRs that recognize the DNA and RNA-containing immune complexes using LSG biopsy samples from 32 SjD patients and 18 non-SjD patients (**Figure 1I**). The results showed statistically significantly higher expression of *TLR7* and *TLR8* in the SjD group compared with the HC group.

### 3.4 Infiltration of *TLR8*-expressing macrophages and monocytes in LSGs

**Figure 2:**
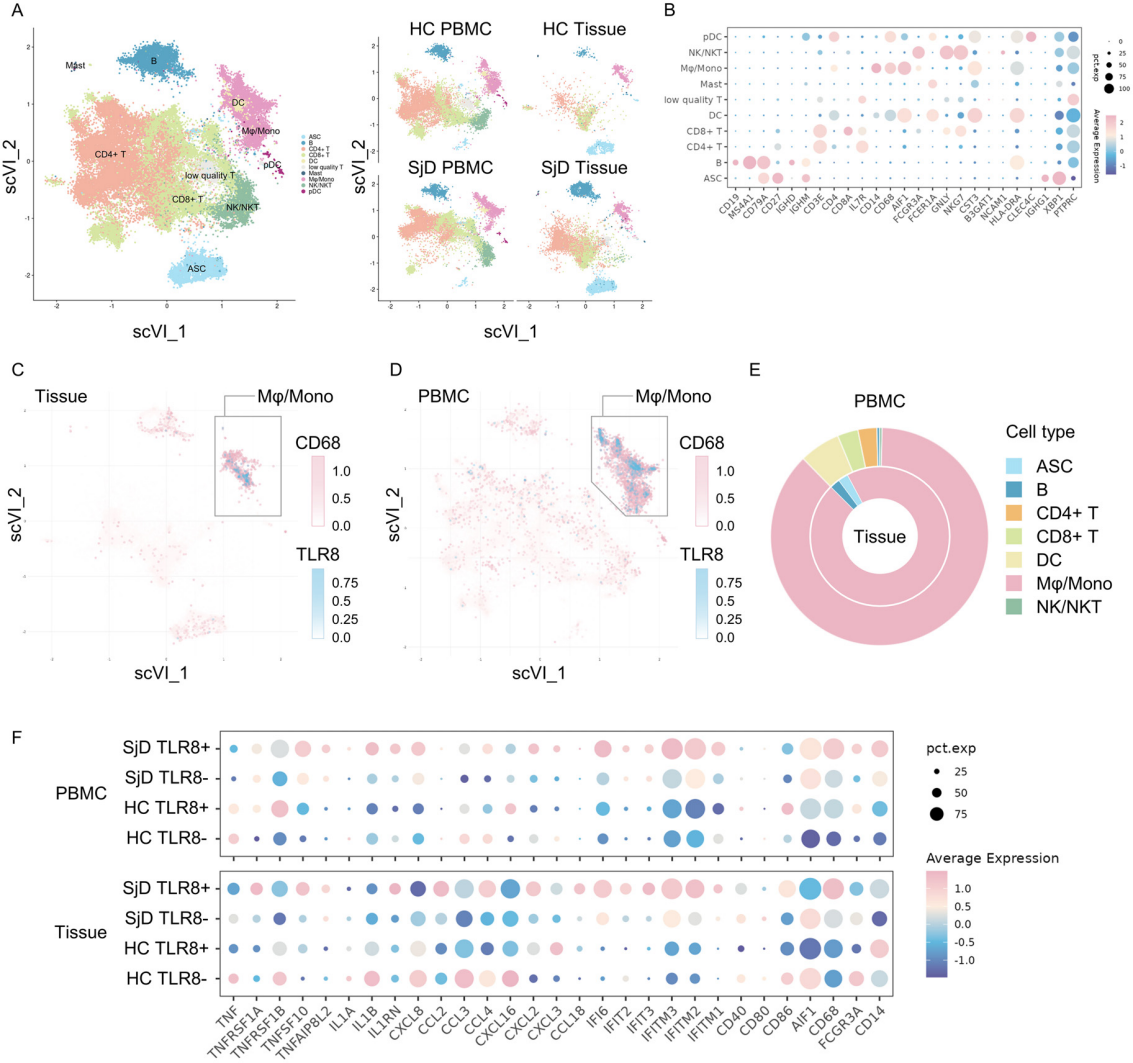
Single-cell RNA-seq analysis for both tissue infiltrating and circulating macrophages/monocytes. **(A)** Visualizations of immune cell subsets. A total of 84,017 cells from 27 samples are shown entirely (left) and divided by groups and source (right). ASC: antibody-secreting cells, B: B cells, CD4^+^ T: cluster of differentiation 4^+^ T cells, CD8^+^ T: CD8^+^ T cells, DC: dendritic cells, Mast: mast cells, Mφ/mono: macrophages/monocytes, NK/NKT: natural killer cells/natural killer T cells, pDC: plasmacytoid dendritic cells. **(B)** Expression of marker genes for identifying cell phenotypes. The color reflects the average expression after normalization, and the size reflects the percentage of markers expressed in these clusters. **(C, D)** Feature plot of *CD68* and *TLR8* expression. *TLR8*-expressing cells are clearly concentrated in the *CD68* ^high^ Mφ/mono cluster both in tissues and PBMCs. **(E)** Composition of *TLR8*-expressing cells. The proportion of *TLR8*-expressing cells in the Mφ/mono cluster was markedly higher than those of other cell clusters, whether in tissues (inner) or PBMCs (outer). **(F)** Gene expression patterns of the different groups. Eight groups were classified by SjD/HC group, tissue/PBMC sample, and *TLR8* ^+/−^ status. The gene expression patterns related to pro-inflammation and T cell activation, chemokines, IFN response, and MHC I and MHC II antigen processing are shown. RNA-seq, RNA sequence; TLR, Toll-like receptor; Mφ/mono, macrophages/monocytes; PBMC, peripheral blood mononuclear cell; SjD, Sjögren’s disease; HC, healthy controls; IFN, interferon; MHC, major histocompatibility complex.

Because only *TLR8* was up-regulated in SjD in both the DNA microarray and real-time PCR analyses, we decided to investigate TLR8 and its source in more detail. To identify the *TLR8*-expressing cells and understand the characteristics and functions of these cells, we integrated our scRNA-seq data with published data, selected immune cells, and removed the potential batch effect using the scVI algorithm, an integration tool based on deep learning (**Figure 2A**). Various subsets of immune cells, such as lymphocytes, DCs, plasmacytoid DCs, antibody-secreting cells, and Mφ/monocytes were identified on the basis of the marker genes (**Figure 2B**). A Mφ/mono subset with high expression of *CD14*, *CD68*, and *AIF1* was identified in LSG tissue and blood in both the SjD and HC groups, revealing the widespread presence of innate immune cells, such as phagocytes. We also investigated the *TLR8* expression levels in these immune cells (**Figures 2C, D**). The vast majority of *TLR8-*expressing cells were located in the Mφ/mono subset, merging with cells that highly expressed *CD68.* Additionally, the proportion of the Mφ/mono subset in *TLR8*-expressing cells far exceeded that of other subsets (**Figure 2E**). These results were consistent in both the tissue and PBMC data, indicating that the main cell populations expressing *TLR8* in humans are macrophages and monocytes, and these cells also frequently infiltrate LSGs.

### 3.5 Distinctive gene expression pattern of *TLR8*-expressing Mφ/mono in SjD tissues

The activation of the TLR signaling pathway can drive different gene expression patterns to promote tissue inflammation in antigen-presenting cells. We divided these cells in the Mφ/mono subset into four groups by SjD or HC group, PBMC or tissue sample, and whether *TLR8* was expressed. We then compared the gene expression patterns using several gene sets, such as pro-inflammation and T cell activation, chemokine, IFN response, major histocompatibility complex (MHC) I pathway, and MHC II pathway (**Figure 2F**). The *TLR8*-expressing Mφ/mono subset in SjD tissue demonstrated a distinctive gene expression pattern compared with other Mφ/mono subsets. These cells had up-regulated expression of genes related to chemokines, responses to IFNs, and MHC I and II antigen processing and presentation pathways compared with the cells from the tissue *TLR8* ^−^ subset in the SjD group and the cells from the tissue *TLR8* ^+^ subset in the HC group. These results suggested enhanced pro-inflammatory and autoimmune responses, as well as strengthened antigen processing and presentation for T cells to activate the subsequent adaptive immune response in SjD. Interestingly, the cells from the PBMC *TLR8* ^+^ subset in the SjD group exhibited high *IL1B* expression, whereas no significantly high expression of *TNF* was detected in both *TLR8* ^+^ and *TLR8* ^−^ macrophages and monocytes from the SjD group in our scRNA-seq analysis.

### 3.6 Enhanced infiltration of TLR8^+^ macrophages in SjD patients compared with CS patients

**Figure 3:**
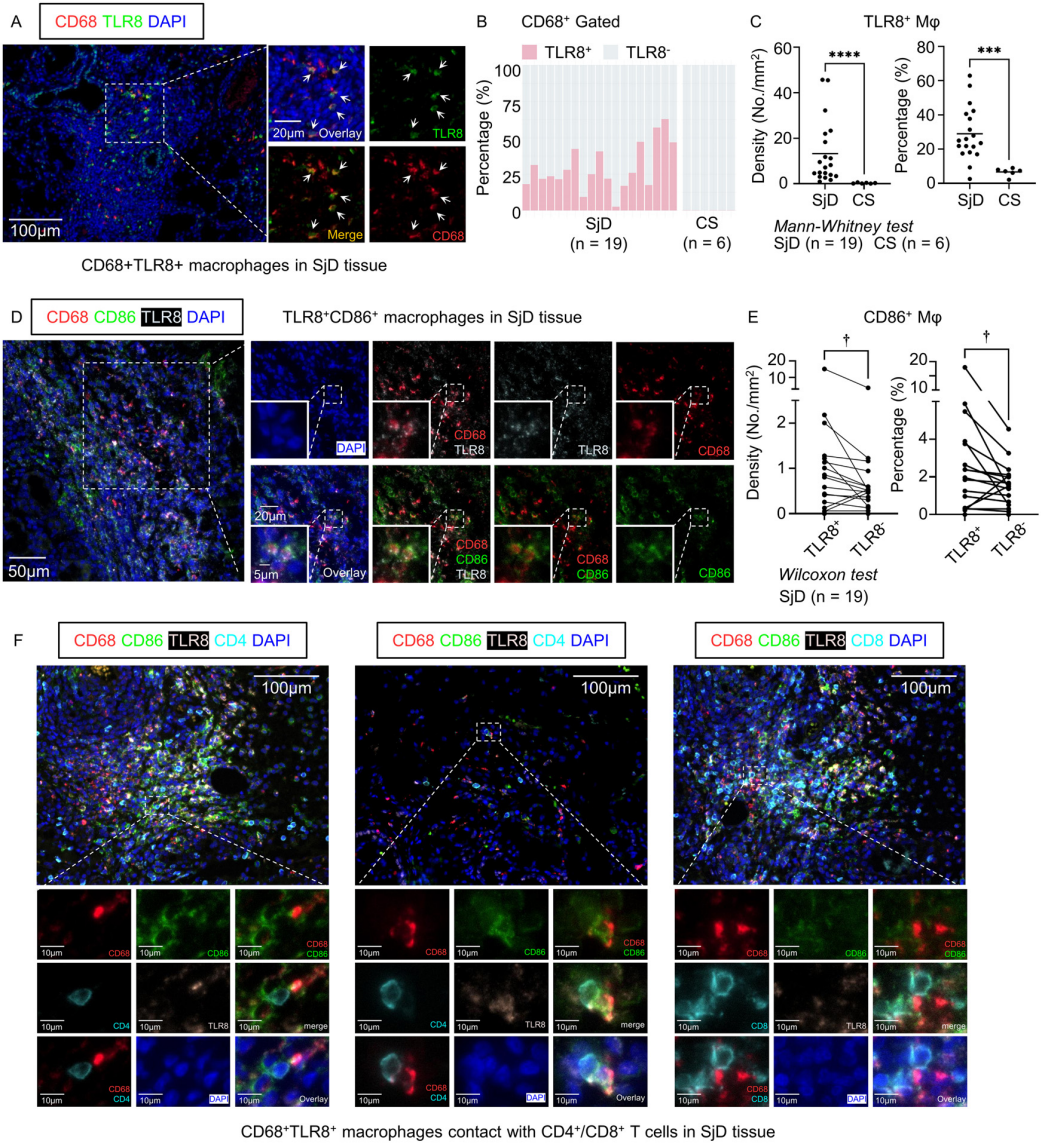
Multi-color immunofluorescence staining and cell quantification. **(A)** Representative images of CD68^+^ TLR8^+^ macrophages in SjD tissue. Scale bars: 100 µm (low magnification) and 20 µm (high magnification). **(B)** Composition ratio of TLR8^+/−^ macrophages in SjD (n = 19) and CS (n = 6) tissues, gated by CD68^+^. **(C)** Density and percentage of TLR8^+^ macrophages in SjD and CS tissues. **(D)** Representative images of TLR8^+^ CD86^+^ macrophages in SjD tissue. Scale bars: 50 µm (low magnification), 20 µm (medium magnification), and 10 µm (high magnification). **(E)** Density and proportion of CD86^+^ expression in TLR8^+/−^ macrophages in SjD tissue. Statistically significant differences between groups were determined by Mann–Whitney *U* test (****P < 0.0001, ***P < 0.001) and Wilcoxon matched-pairs signed rank test (†*P* < 0.05). **(F)** Representative images of TLR8^+^ CD86^+^ macrophages contact with CD4^+^ or CD8^+^ T cells in SjD tissue. Scale bars: 50 µm (low magnification) and 10 µm (high magnification). CD, cluster of differentiation; TLR, Toll-like receptor; SjD, Sjögren’s disease; CS, chronic sialadenitis.

Given the distinctive gene expression pattern and intriguing function of *TLR8*-expressing macrophages shown in the microarray and scRNA-seq analyses, we screened LSG tissues from SjD patients and SMG tissues from CS patients with CD68^+^ macrophage infiltration and performed multi-color IF staining using these tissue sections from SjD (n = 19) and CS (n = 6) patients. These tissue sections were then stained with CD68 for macrophages, with TLR8 staining (**Figure 3A**). The proportion of TLR8^+^ macrophages in the SjD tissues greatly exceeded that in CS tissues (**Figure 3B**), which is a nonspecific salivary gland inflammation and often a result of ductal stricture and obstruction. We further compared the proportion of TLR8^+^ macrophages in all macrophages and the density of TLR8^+^ macrophages between SjD and CS tissues. The results showed that both the proportion and density of TLR8^+^ macrophages in SjD tissues were significantly higher than those in CS tissues (**Figure 3C**), indicating a specific role of TLR8 in autoimmune diseases, such as SjD. In addition, to classify if disease progression affects the expression of TLR8 in macrophages, we investigated the correlation between symptom duration and density and percentage of TLR8-positive monocytes/macrophages respectively, which presented a negative correlation but without statistically significant (**Supplementary Figure 2**).

### 3.7 TLR8^+^ macrophages have high expression of CD86 in SjD tissues

The scRNA-seq analysis showed that the cells in the *TLR8*-expressing Mφ/mono subset infiltrating SjD tissues contained high expression of *CD86* and genes in the MHC antigen processing pathway. To further verify the potential correlation of CD86 and TLR8 in macrophages and considering the crucial role of other B7 molecules, such as CD80, in activating T lymphocytes and initiating the adaptive immune response, we also stained SjD tissue sections, as mentioned, with CD86 and CD80 (**Figure 3D**, **Supplementary Figure 3A**). Consistent with the results of the scRNA-seq analysis, when we paired the TLR8^+^ and TLR8^−^ macrophage groups from the same tissue section and compared the frequency of CD86^+^ cells in the two groups, TLR8^+^ macrophages had a significantly higher positivity rate for CD86 (**Figure 3E**). Given that the expression level of *CD80* in the Mφ/mono subset was very low in the scRNA-seq analysis, which may not reflect the situation in SjD tissues completely, we also analyzed CD80 expression at the protein level. Although CD80^+^ macrophages were rare in SjD tissue sections, consistent with CD86, TLR8^+^ macrophages had higher expression of CD80 compared with TLR8^−^ macrophages (**Supplementary Figure 3**). These findings suggest that TLR8 activation is potentially related to the enhancement of B7 molecule expression, such as CD86 and CD80; thereby, providing a secondary signal for T cell activation. In fact, CD86^+^ TLR8^+^ macrophages frequently interacted with CD4^+^ T cells even in areas with sparse lymphocyte infiltration, and in regions with extensive lymphocyte infiltration, they also frequently engaged with CD8^+^ T cells in addition to CD4^+^ T cells (**Figure 3F**).

## 4 Discussion

TLR7 and TLR8 can seek bacterial RNA by locating the RNA in phagocytic endosomes. When they are activated, NF-κB, activating protein 1, and IFN regulatory factor mediate the production of type I IFN, which results in the production of pro-inflammatory cytokines (29). TLR7 and TLR8 are phylogenetically very close. As a result, the natural ligand single-stranded RNA produced by viruses stimulates both TLR7 and TLR8 in humans. There is a wealth of circumstantial evidence supporting enhanced TLR7 signaling as a mechanism for human systemic autoimmune diseases (30, 31). In comparison, TLR8 is considered non-functional in mice and has not been studied as much as TLR7; hence, TLR8 is one of the least-studied members of the TLR family (32). Demaria et al. generated TLR8-deficient mice through gene targeting and revealed that mouse TLR8 plays a pivotal role in the regulation of mouse TLR7 expression and the spontaneous autoimmunity (33). If TLR8 regulates TLR7 expression upstream of TLR7 in humans, the association of TLR8 with autoimmune disease pathogenesis may have been underestimated and should be investigated. SjD is an intractable chronic systemic autoimmune disease with no established treatment. Although TLR8 has not been investigated in the pathogenesis of SjD, there are scattered reports of a crucial role for TLR7 signaling in the local and systemic manifestations of disease in SjD, and inhibition of such will likely have therapeutic value (9, 34, 35).

In our study, a comprehensive broad investigation of immune cells in SjD tissues identified significantly high expression of innate immunity-related transcripts, including TLRs. Consistent with previous reports, *TLR7* was up-regulated only in SjD in the present study. Notably, a comprehensive unbiased approach using affected organs in SjD showed that *TLR8* was also up-regulated only in SjD. Furthermore, the increase in *TLR8* expression was more pronounced than that of *TLR7* in the gene set enrichment analysis, where the TLR signaling pathway showed significant enrichment and was up-regulated, overall. With up-regulation of *TLR8* expression, the transcription levels of many molecules in the NF-κB signaling pathway, which is considered a representative signaling pathway of *TLR8*, were also up-regulated.

In the scRNA-seq analysis, *TLR8* was predominantly expressed by monocytes and macrophages in the tissues and PBMCs, in patients with SjD. *TLR8* ^+^ cells in the monocyte/macrophage cluster exhibited high expression of pro-inflammatory cytokines and chemokines, as well as genes related to the MHC antigen processing pathway compared with *TLR8*-negative cells in that cluster. While a direct pathogenic role for these cytokines via the *TLR8* signaling pathway is still questionable, *TLR8*-positive cells in the monocyte/macrophage cluster may also contribute to the pathogenesis of SjD. It is well documented that viruses, including Epstein–Barr virus, hepatitis C virus, human T cell lymphotropic virus type 1, and cytomegalovirus, can influence the immune system and initiate autoimmune reactions through various mechanisms (36, 37). Considering the many reports of viral and bacterial infections, especially Epstein–Barr virus, as triggers for the pathogenesis of SjD, it is conceivable that TLR8-mediated recognition of viral or bacterial single-stranded RNA could trigger the production of inflammatory cytokines, leading to the development of SjD (36). Quantitative investigation of SjD tissues in this study directly demonstrated striking infiltration of TLR8-positive macrophages and CD86 expression in these cells. Considering that TLR8-positive macrophages were rarely observed in CS, which is a nonspecific inflammation, TLR8 molecules and the macrophages that express this molecule likely contribute to the specific pathogenesis of SjD via the activation of an adaptive immune response mediated by T lymphocytes. Therefore, the roles of these cells in SjD, as well as in other autoimmune diseases, should be extensively investigated in the future.

We previously showed that SjD is largely driven by a type I immune response dominated by CD8^+^ cytotoxic T lymphocytes and the apoptotic killing of cellular self-targets, with a relative abundance of Th1 and T follicular helper cells, in SjD tissues (3). The present data indicate that TLR8-positive monocytes/macrophages activate CD8^+^ cytotoxic T lymphocytes by antigen cross-presentation through the MHC I pathway and CD4^+^ Th cells by antigen presentation through the MHC II pathway, as shown in both the scRNA-seq analysis and multi-color IF staining results. Given that our observations of an activated signaling pathway via up-regulation of several molecules and those of recent reports, TLR8-positive monocytes/macrophages may activate both CD8^+^ and CD4^+^ T cells via antigen presentation and B7 molecules binding with CD28 on T cells. However, in areas with sparse lymphocyte infiltration, contact between CD8^+^ T cells and CD86^+^ TLR8^+^ macrophages was rare compared to CD4^+^ T cells, suggesting that CD4^+^ T cells are more involved from the initiation stage of SjD pathogenesis, while CD8^+^ T cells may play a more prominent role at a slightly later stage of that. This process may contribute to Th1 preferential differentiation by producing TNFα and IFNγ, as well as T follicular helper preferential differentiation by producing interleukin-6 (38–40).

**Figure 4:**
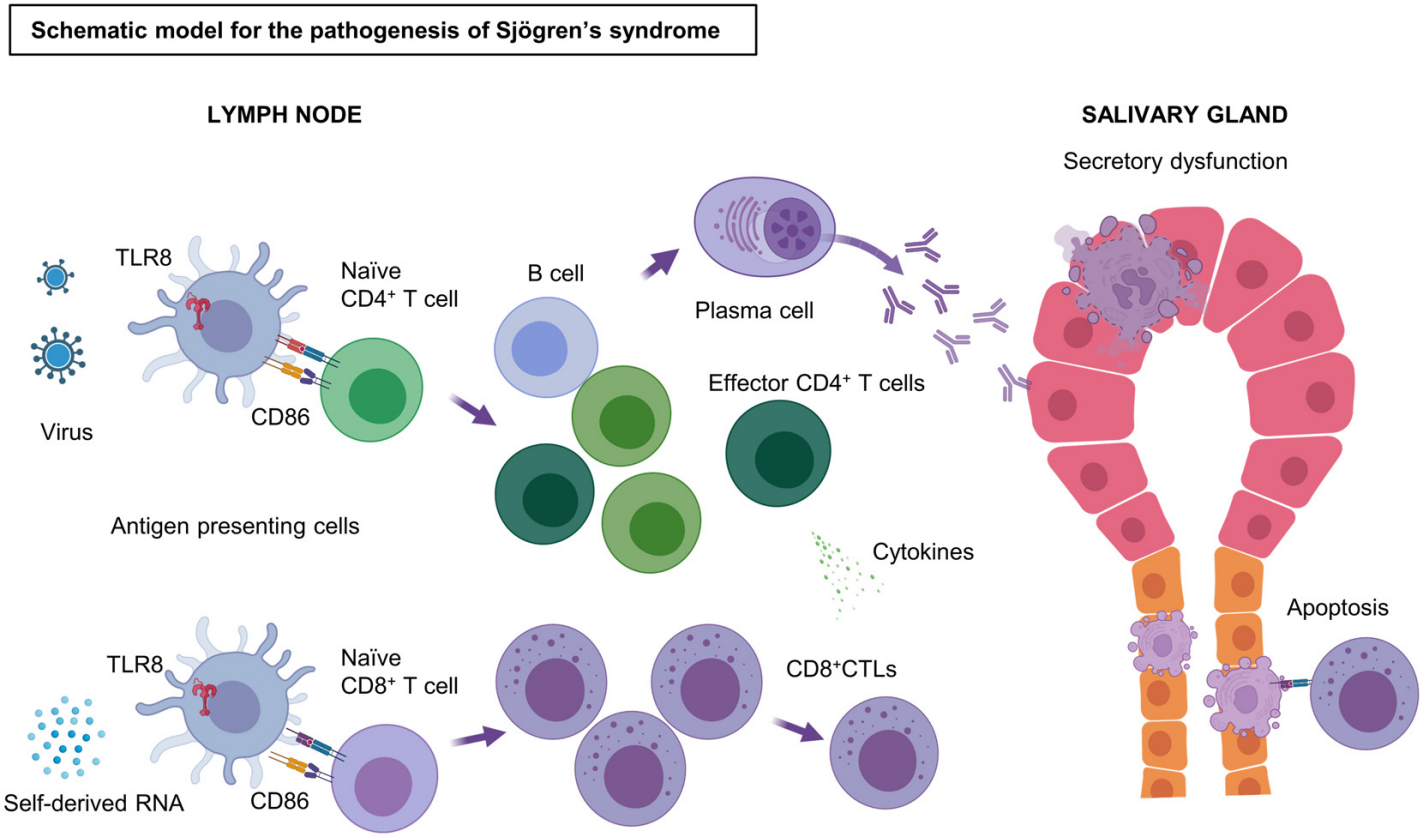
Schematic model of the pathogenesis of SjD. Stimulation by antigens, such as self-derived RNA and viruses, triggers and activates the TLR8 signaling pathway in TLR8^+^ monocytes/macrophages. These TLR8^+^ monocytes/macrophages strongly express CD86 and induce T cell activation by binding CD28 on T cells. CD4^+^ Th cells are activated by antigen presentation through the MHC II pathway, whereas Naïve CD8^+^ T cells become effector CD8^+^ CTLs by antigen cross-presentation through the MHC I pathway. CD8^+^ CTLs cause apoptosis of salivary gland cells via their cytotoxicity. Some of the effector CD4^+^ T cells may help in the differentiation of B cells to plasma cells, which produce antibodies to the autoantigens, resulting in irreversible secretory dysfunction. TLR, Toll-like receptor; CD, cluster of differentiation; CTL, cytotoxic T lymphocytes; MHC, major histocompatibility complex; Th, T helper cells. Figure created with BioRender.com.

In conclusion, our experiments indicate that the TLR8 signaling pathway, which is readily induced in the altered, possibly virus-infected milieu generated in tissue, causes specific pathologies in SjD. We also showed that TLR8-positive monocyte/macrophages exhibited up-regulation of chemokines, MHC antigen processing, and B7 molecules. These processes are likely to create an immune environment conducive to dominance of effector CD8^+^ and CD4^+^ T cells and adaptation and initiation of humoral immune responses. These specific environments subsequently cause apoptosis of ductal cells and acinar cells in the salivary gland, as well as the production of autoantibodies via autoreactive B cells. A schematic overview of the pathogenesis of SjD on the basis of our observations and speculations is presented in **Figure 4**. This potential disease initiation mechanism would be consistent with the prominent secretory dysfunction and xerostomia observed in patients with SjD. Our findings suggest that the association between TLR8 and the pathogenesis of SjD is underappreciated. The present analysis heightens our understanding of TLR8 in the pathogenesis of SjD. However, our study also has limitations. First, only a few samples were analyzed owing to the difficulty of extracting immune cells from the minor salivary gland tissue. Additionally, the lack of functional approach using cell culture techniques or animal experiments has led to an incomplete understanding of the role of TLR8 in the pathogenesis of SjD. Thus, TLR8-positive monocyte/macrophages should be further investigated as both a potential driver and a therapeutic target in SjD.

## Data Availability

The datasets generated during and/or analysed during the current study are available from the corresponding author on request. Sequence data presented in the study are deposited in National Center for Biotechnology Information GEO database (https://www.ncbi.nlm.nih.gov/geo/), accession number GSE279818.
