# Supplementary material for: Possible involvement of Toll-like receptor 8-positive monocytes/macrophages in the pathogenesis of Sjögren’s disease
Source: Front Immunol. 2024 Oct 31;15:1480675. doi: 10.3389/fimmu.2024.1480675 (PMC11560440; doi:10.3389/fimmu.2024.1480675)
Supplement: Supplementary file 1 [file DataSheet1.pdf]

Supplementary Table 1: Clinical data on primary Sjögren's disease patients studied

Patient	Sex	Age	Symptom Duration (year)	Serological Tests								
				RF	ANA	Anti DNA	Anti SS-A	Anti SS-B	High IgG	High IgA	High IgM	High IgG4
P01	F	92	9	-	+	-	+	-	+	-	-	NT
P02	F	62	7	+	+	-	+	-	-	-	-	NT
P03	F	48	1	+	+	NT	+	+	-	-	-	-
P04	F	80	2	NT	NT	NT	+	-	NT	NT	NT	NT
P05	F	52	3	+	+	NT	+	+	-	-	-	NT
P06	F	70	8	+	+	-	+	-	-	+	-	-
P07	F	66	12	NT	NT	NT	-	-	NT	NT	NT	NT
P08	F	73	12	NT	NT	NT	+	-	NT	NT	NT	NT
P09	F	65	2	+	+	NT	+	+	+	-	-	-
P10	F	52	11	+	+	-	+	+	+	-	-	NT
P11	F	72	13	-	±	-	+	-	NT	NT	NT	NT
P12	F	56	14	+	+	-	+	+	-	-	-	NT
P13	F	74	10	NT	NT	NT	+	+	NT	NT	NT	NT
P14	F	59	29	+	NT	NT	+	+	NT	NT	NT	NT
P15	F	81	7	+	+	NT	+	+	+	-	-	NT
P16	F	81	14	+	+	-	+	+	-	-	-	NT
P17	F	74	21	-	+	NT	-	-	-	-	-	NT
P18	F	80	14	NT	+	-	+	+	+	+	-	NT
P19	F	66	9	-	+	-	+	+	+	-	-	NT
P20	F	60	10	-	+	NT	-	-	-	-	-	NT
P21	F	71	13	-	+	-	+	-	-	+	-	NT
P22	F	72	16	NT	NT	NT	+	-	NT	NT	NT	NT
P23	F	87	22	-	+	-	-	-	-	-	-	NT
P24	F	66	7	+	+	-	+	-	-	-	-	NT
P25	F	81	12	NT	NT	NT	+	-	NT	NT	NT	NT
P26	F	49	7	-	+	NT	+	+	+	-	-	-
P27	F	70	10	-	+	-	+	NT	NT	NT	NT	NT
P28	F	54	13	-	+	-	+	-	NT	NT	NT	NT
P29	F	73	10	NT	NT	NT	+	+	NT	NT	NT	NT
P30	F	48	9	+	+	-	+	-	+	-	-	NT
P31	F	69	17	+	NT	NT	+	+	+	-	-	NT
P32	F	79	12	NT	NT	NT	+	+	NT	NT	NT	NT
P33	F	53	11	+	+	-	+	+	NT	NT	NT	NT
P34	F	66	12	NT	NT	NT	+	-	NT	NT	NT	NT
P35	F	53	9	+	+	NT	+	+	NT	NT	NT	NT
P36	F	84	11	NT	NT	NT	+	+	NT	NT	NT	NT
P37	F	81	7	NT	+	-	+	-	+	-	-	NT
P38	F	16	4	+	+	+	+	+	+	-	-	-
P39	F	56	6	NT	NT	NT	+	+	NT	NT	-	-
P40	F	83	6	NT	NT	NT	+	-	NT	NT	NT	NT
P41	F	30	3	+	+	NT	+	-	+	-	-	NT
P42	F	42	6	+	+	NT	+	+	+	NT	NT	NT
P43	F	65	14	-	+	-	+	NT	-	-	-	NT
P44	F	73	11	NT	-	NT	-	-	NT	NT	NT	NT
P45	F	76	5	-	+	NT	-	-	-	-	-	NT
P46	F	82	4	NT	NT	NT	+	-	NT	NT	NT	NT
P47	F	72	3	+	+	-	+	+	+	+	-	NT
P48	F	50	6	+	+	NT	+	+	+	+	+	NT
P49	F	72	6	+	+	-	+	+	-	-	-	NT
P50	F	75	6	+	+	NT	+	-	-	-	-	NT
P51	F	16	4	±	+	+	+	+	+	-	-	NT
P52	F	78	6	-	+	NT	+	+	-	+	-	NT
P53	F	77	2	+	NT	NT	+	+	+	+	-	-

Reference value of serological tests: RF: < 15 mg/dL; ANA: < 40 times; Anti DNA: < 10.0 IU/mL; SSA: < 7.0 U/mL; SSB: < 7.0 U/mL; IgG: 1747-861 mg/dL; IgA: 393-93 mg/dL; IgM: 183-33 mg/dL; IgG4: 121-11 mg/dL. RF: Rheumatoid factor; NT: not tested.

(Continuation sheet)

Patient	Salivary Flow Rate			Sialography	Scintigraphy	Pathology	Schirmer's Test	Application
	Spitting method (mL/15min)	Saxon test (g/2min)	Gum test (mL/10min)					
P01	0	2.2	12.2	+ (Stage I)	+	+	-	DNA microarray
P02	0.1	0.53	3.2	+ (Stage III)	+	+	NT	DNA microarray
P03	0.1	1.57	0.6	NT	NT	++	-	DNA microarray
P04	0.01	0.23	NT	Stage 0	-	+	NT	DNA microarray
P05	0.6	1.63	6.2	+ (Stage I)	+	++	-	DNA microarray
P06	0.38	3.1	9.05	+ (Stage III)	+	+	NT	DNA microarray, RT-PCR
P07	1.9	0.3	7.5	Stage 0	-	++	NT	RT-PCR
P08	0.6	0.66	7	+ (Stage I)	+	+	+	RT-PCR
P09	0.1	0.21	2.5	+ (Stage III)	+	+	NT	RT-PCR
P10	0.59	1.95	9.31	+ (Stage II)	+	+	NT	RT-PCR
P11	0.25	1.13	5.6	+ (Stage I)	-	NT	NT	RT-PCR
P12	0.12	0.83	7.8	+ (Stage III)	+	+++	NT	RT-PCR
P13	0.05	0.49	3.6	+ (Stage II)	+	++	NT	RT-PCR
P14	0.18	0.98	5.46	+ (Stage II)	+	+	NT	RT-PCR
P15	0	1.99	1.53	+ (Stage I)	-	+	NT	RT-PCR
P16	0.52	2.2	9	+ (Stage III)	+	+	NT	RT-PCR
P17	0.08	0.3	1.8	+ (Stage I)	+	++	NT	RT-PCR
P18	1.96	1.51	11.85	+ (Stage I)	+	+	NT	RT-PCR
P19	0.3	1.57	2.6	Stage 0	+	+	-	RT-PCR
P20	0	0.64	2	+ (Stage II)	-	+	NT	RT-PCR
P21	0.2	0.27	1.5	+ (Stage IV)	+	+++	-	RT-PCR
P22	0.43	0.71	8.2	+ (Stage I)	-	++	NT	RT-PCR
P23	0.6	0.96	6.2	+ (Stage II)	+	+	-	RT-PCR
P24	0.03	0.3	1.36	+ (Stage IV)	+	+	+	RT-PCR
P25	0.5	2.88	8.8	+ (Stage II)	NT	+	NT	RT-PCR
P26	11	5.42	8.8	+ (Stage I)	+	++	+	RT-PCR
P27	0.9	1.72	2.8	+ (Stage I)	+	+	NT	RT-PCR
P28	1.5	3.01	9.8	+ (Stage II)	NT	++	NT	RT-PCR
P29	0.1	1.6	7.6	Stage 0	+	+	NT	RT-PCR
P30	1.2	1.38	9.6	+ (Stage II)	+	+	NT	RT-PCR
P31	0.5	1.7	6.1	+ (Stage II)	+	++	NT	RT-PCR
P32	1	1.71	8.8	+ (Stage II)	+	+++	NT	RT-PCR
P33	1.52	1.95	8.84	+ (Stage I)	+	+	+	RT-PCR
P34	0.18	1.32	4.1	+ (Stage II)	+	+++	NT	RT-PCR, multi-color IF
P35	2	2.02	16	+ (Stage III)	+	+	NT	RT-PCR, multi-color IF
P36	0	0.59	3.2	+ (Stage II)	+	±	-	RT-PCR, multi-color IF
P37	0.85	6.48	10.18	+ (Stage II)	-	+	NT	RT-PCR, multi-color IF
P38	0.9	1.23	4.7	+ (Stage II)	-	++	-	multi-color IF
P39	1	1.83	13	+ (Stage I)	+	+++	NT	multi-color IF
P40	0	0.14	2	+ (Stage III)	+	+++	-	multi-color IF
P41	1.8	1.99	5.6	+ (Stage I)	+	++	NT	multi-color IF
P42	1.3	5.28	8.5	NT	NT	+	NT	multi-color IF
P43	1.6	1.56	7.1	+ (Stage I)	-	++	NT	multi-color IF
P44	1	1.84	6.8	+ (Stage I)	+	+	NT	multi-color IF
P45	0.4	1.74	2	+ (Stage I)	+	+	-	multi-color IF
P46	NT	0.08	NT	+ (Stage I)	+	±	NT	multi-color IF
P47	0.08	0.2	NT	+ (Stage II)	-	+	-	multi-color IF
P48	1.45	1.2	5.6	+ (Stage III)	+	+	-	multi-color IF
P49	0.1	5.03	8.2	+ (Stage I)	-	+	-	multi-color IF
P50	0.1	0.21	1.3	+ (Stage III)	+	+	-	multi-color IF
P51	3	2.9	19	+ (Stage I)	-	+	-	multi-color IF
P52	0.64	3.74	6.92	+ (Stage I)	-	+	-	multi-color IF
P53	1.9	3.25	13.2	+ (Stage II)	+	+	NT	scRNA-seq

Spitting method positive: ≤ 1.5mL/15min; Saxon test positive: ≤ 2g/2min; Gum test positive: ≤ 10mL/10min. Stage classification of sialography: Stage 0: normal; Stage I: punctate; Stage II: globular; Stage III: cavitary; Stage IV: destructive. Classification of pathology: -: no change; ±: very slight; +: minor; ++: moderate; +++: advanced. Schirmer's Test positive: ≤ 5mm/5min. NT: not tested; RT-PCR: real-time polymerase chain reaction; multi-color IF: multi-color immunofluorescence staining; scRNA-seq: single-cell RNA sequencing.

Supplementary Table 2: Primers of real-time PCR

Gene	PCR product size (bp)	Primer sequence	
**TLR7**	262	forward	5’- TGG AAA TTT TGG ACC TCA GC -3’
		reverse	5’- TTG CAA AGA AAG CGA TTG TG -3’
**TLR8**	195	forward	5′- AAG CAC ATC CCA AAT GAA GC -3′
		reverse	5′- GCA ACT CGA GAC GAG GAA AC -3′
**TLR9**	265	forward	5’- CAA TGT CAC CAG CCT TTC CT -3’
		reverse	5’- GCT GAG GGA CAG GGA TAT GA -3’
**β-actin**	260	forward	5′- GCA AAG ACC TGT ACG CCA AC -3′
		reverse	5′- CTA GAA GCA TTT GCG GTG GA -3′

Supplementary Figure 1

A

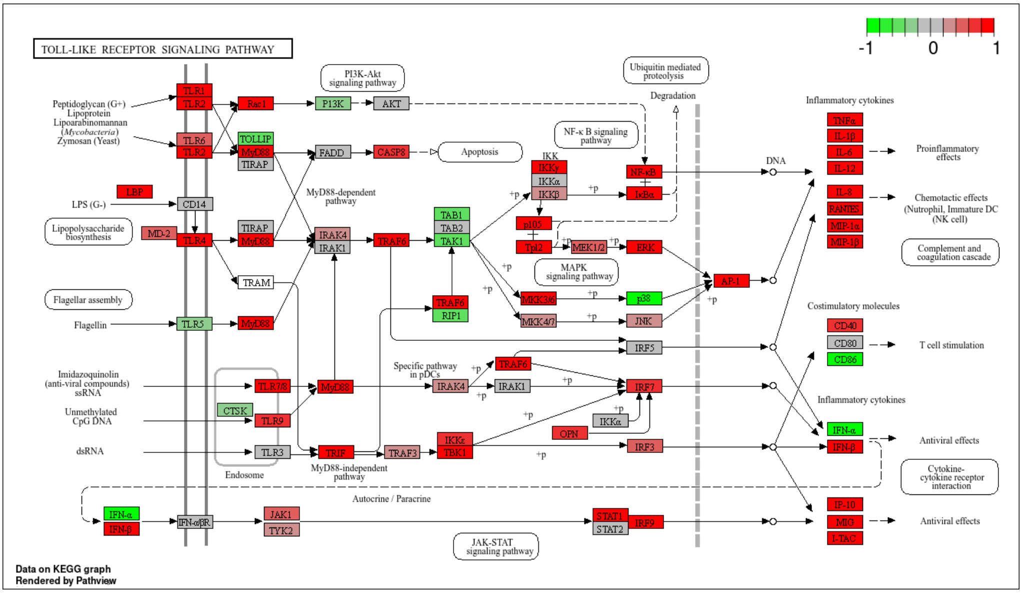

B

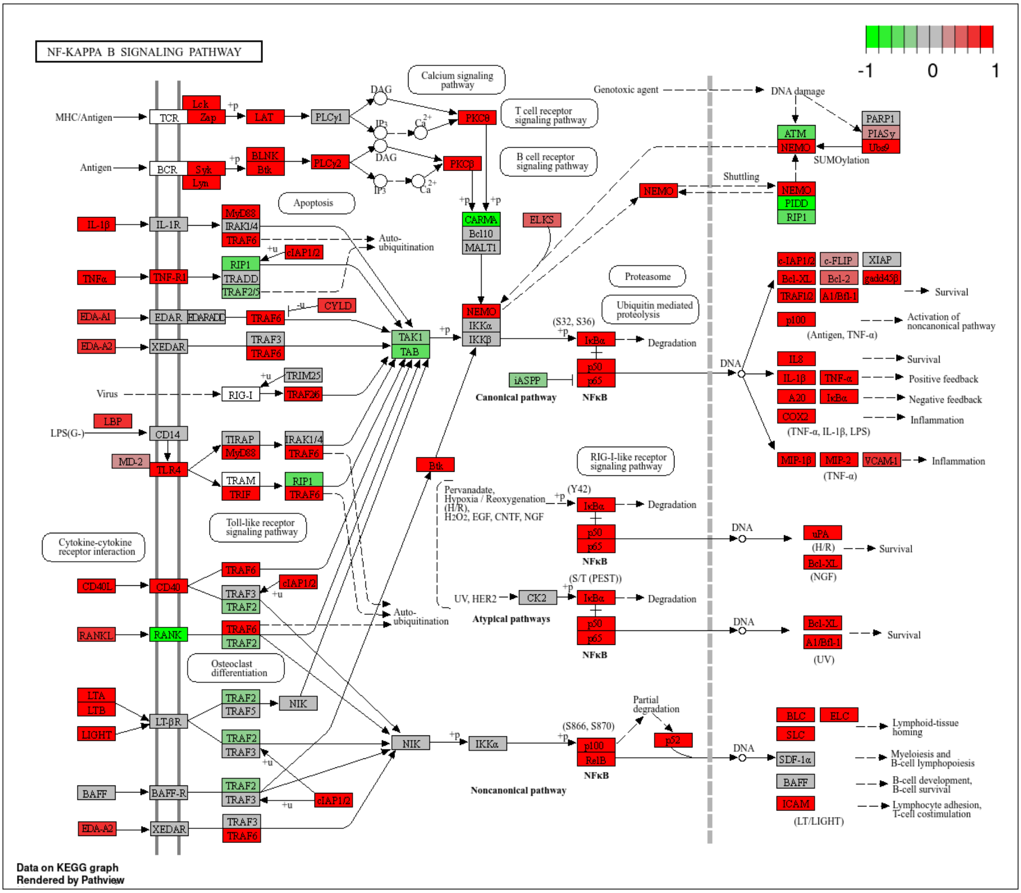

Supplementary Figure 1. Gene set enrichment analysis. Enriched genes are highlighted in KEGG toll-like receptor signaling pathway **(A)** and NF-κB signaling pathway **(B)**.

Supplementary Figure 2

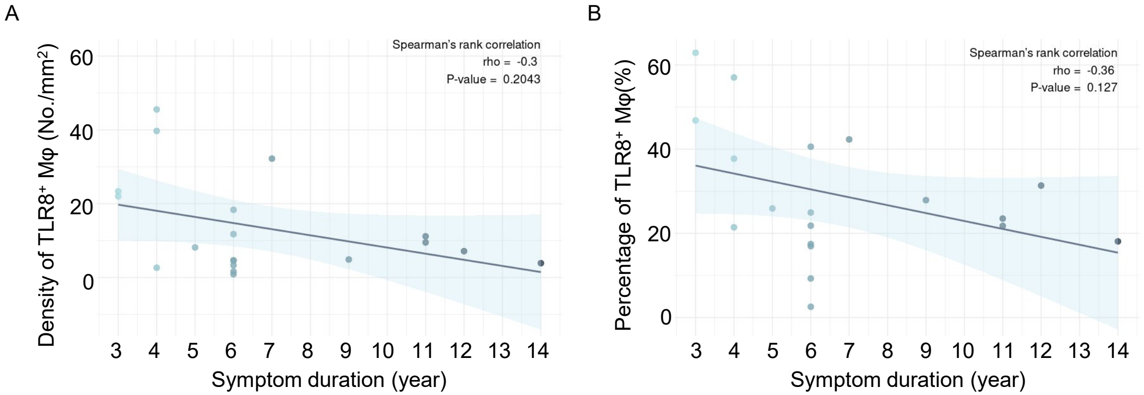

Supplementary Figure 2. Scatter plots of the Spearman's rank correlation between symptom duration and **(A)** density or **(B)** percentage of $TLR8^{+}$ macrophages, showing a negative correlation in both cases (rho < 0), but these correlations were not statistically significant (*p*-values > 0.05).

Supplementary Figure 3

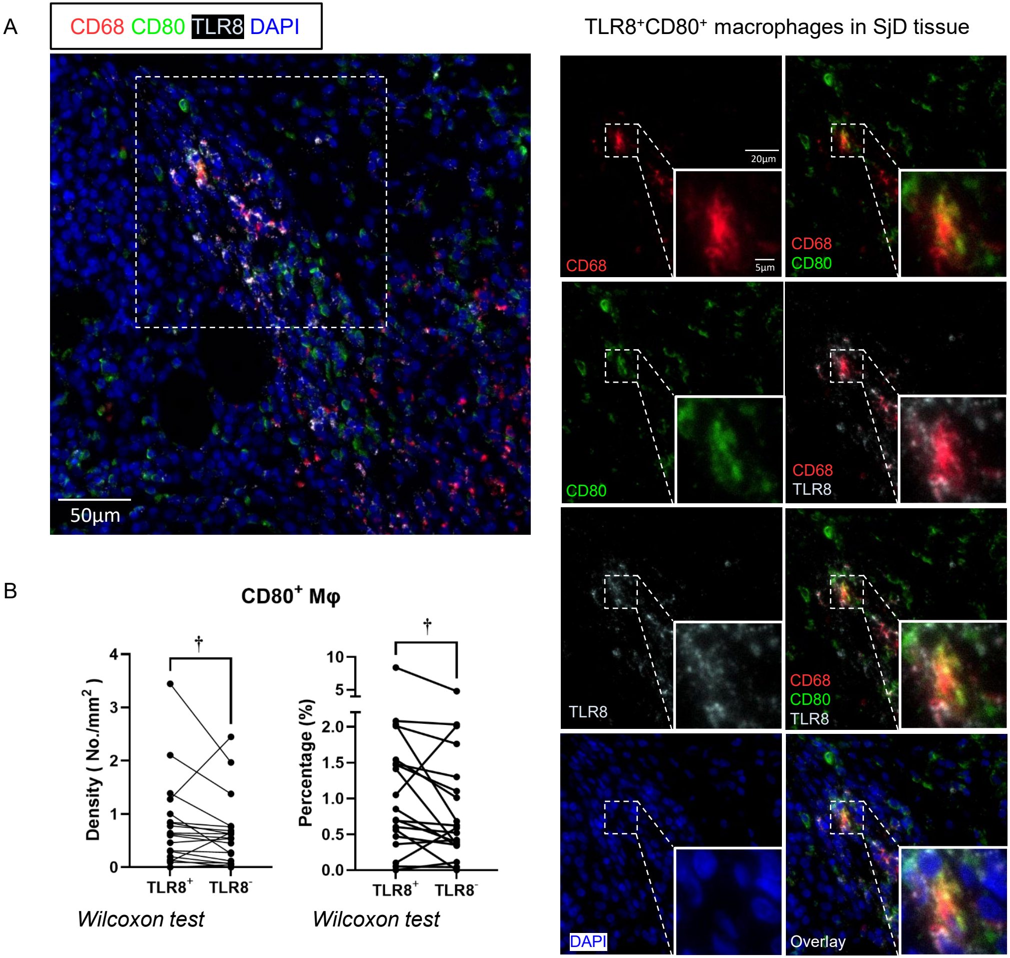

Supplementary Figure 3. Multicolor immunofluorescence staining and cell quantification. **(A)** Representative images of TLR8⁺CD80⁺ macrophages in SS tissue. Scale bars: 50 µm (low magnification), 20 µm (medium magnification), 10 µm (high magnification). **(B)** Density and proportion of CD80⁺ in TLR8$^{+/-}$ macrophages. Statistically significant differences between groups were determined by Wilcoxon matched-pairs signed rank test (†$P < 0.05$).
